# Protective Effect of Red Rice Extract Rich in Proanthocyanidins in a Murine Colitis Model

**DOI:** 10.3390/biomedicines11020265

**Published:** 2023-01-18

**Authors:** Napapan Kangwan, Sarawut Kongkarnka, Komsak Pintha, Chalermpong Saenjum, Maitree Suttajit

**Affiliations:** 1 Division of Physiology, School of Medical Sciences, University of Phayao, Phayao 56000, Thailand; 2 Department of Pathology, Faculty of Medicine, Chiang Mai University, Chiang Mai 50200, Thailand; 3 Division of Biochemistry, School of Medical Sciences, University of Phayao, Phayao 56000, Thailand; 4 Center of Excellence for Innovation in Analytical Science and Technology for Biodiversity-Based Economic and Society (I-ANALY-S-T_B.BES-CMU), Chiang Mai University, Chiang Mai 50200, Thailand

**Keywords:** anti-inflammation, cytokines, inflammatory bowel disease, red rice, dextran sulfate sodium, proanthocyanidins

## Abstract

Inflammatory bowel disease (IBD) has become a global concern. Proanthocyanidin-rich red rice extract (PRRE) has been shown to suppress the inflammatory response in cellular cultures. However, the anti-colitis effect of PRRE has never been investigated in animals. This study aimed to examine the protective effect of the PRRE against dextran sulfate sodium (DSS)-induced colitis in mice. Male mice were orally administrated with PRRE of 50, 250 and 500 mg/kg/day for 21 days. Acute colitis was subsequently induced by administrated 2.5% DSS in drinking water for the final seven days. Sulfasalazine-treated mice were the positive group. All doses of PRRE and sulfasalazine significantly ameliorated DSS-induced severity of colitis, as indicated by decreasing daily activity index and restoring colon shortening. Treatments with PRRE, but not sulfasalazine, significantly reduced the histopathological index and infiltration of inflammatory cells. Furthermore, the PRRE treatments effectively improved mucous in colonic goblet cells using PAS staining, and suppressed the production of pro-inflammatory cytokines TNF-α, IL-1β and IL-6 induced by DSS, while sulfasalazine reduced only IL-1β and IL-6. This study suggested that PRRE had a greater anti-colitis effect than sulfasalazine. Thus, PRRE has a potential anti-colitis effect, and should be developed in a clinical trial as a natural active pharmaceutical ingredient for IBD.

## 1. Introduction

Inflammatory bowel disease (IBD) is a chronic inflammation of the digestive tract, characterized by the continuous conditions of relapsing and remitting. IBD is mainly composed of two subtypes, including Crohn’s disease (CD), which is characterized by the formation of structures, fistulas, ulcers and granulomas in the mucosa in any part of the digestive tract; and ulcerative colitis (UC), which involves mucosal inflammation and ulceration of the colon and rectum [1]. IBD has recently been considered a public health problem affecting patients’ quality of life in Western countries [2]. However, its incidence has grown in Asian countries due to the increased intake of a high-fat, high-sugar and low-fiber diet [3,4]. The pathogenesis mechanism of IBD is not precisely understood. Several factors are involved in the pathogenesis of IBD, including an imbalance of the composition of the intestinal microbiota, dysregulation of the immune system, barrier integrity dysfunction and genetic variations [5]. In accordance with this idea, inflammatory tissue in the animal model and IBD patients has been shown to activate the mucosal immune response, produce oxidative stress and suppress antioxidant defense mechanisms, eventually inducing intestinal damage [6]. In addition, IBD patients have a high risk of gastrointestinal and other cancers resulting from chronic inflammation-induced tumor formation and long-term use of immunosuppressive drugs, such as azathioprine and anti-tumor necrosis factor (TNF) drugs, exerting carcinogenic action [7]. The pharmacological therapy currently available for IBD treatment, such as 5-aminosalicylates, immunosuppressants and corticosteroids, unfortunately has several side effects [8]. Among these drugs, sulfasalazine (Azulfidine) has been used as a standard drug to treat mild to moderate IBD patients. However, it has been shown to induce several adverse effects, such as headache, hepatotoxicity, nephrotoxicity and male infertility [9,10,11]. Therefore, developing alternative medications from natural products with more effectiveness and safety is urgently necessary to treat IBD patients.

Numerous studies have reported that the polyphenol compounds from cereals, vegetables and herbs are potential dietary factors for IBD management [12,13,14,15,16]. Red rice (*Oryza sativa* L.) is a member of the grass family, Poaceae. Red rice is a significant staple food worldwide, particularly in Asian countries, and is also an important economic crop in Thailand. Pigmented rice has grown in popularity, mostly due to its nutritional and health promotional effects. Among pigmented rice, red rice is a predominant source of proanthocyanidins, with strongly exhibited antioxidant, anti-inflammatory, anti-cancer and immunomodulatory activities [17,18,19]. Naturally, red rice accumulates proanthocyanidins in its pericarps, which have potent antioxidant activities and are beneficial to human health [20,21]. In contrast, red yeast rice (Ang-kak), a traditional Chinese food, is white rice fermented by *Monascus purpureus*. Red yeast rice contains metabolites mainly the monacolin family of polyketides [22]. Proanthocyanidins, also known as condensed tannins, are oligomeric and polymeric flavan-3-ols from flavonoid biosynthesis [23]. They mainly include catechin, epicatechin and their 3-O-gallates and epigallates [24]. Proanthocyanidins, having an astringent flavor, are abundant in varieties of the botanical sources and plant food products such as red fruits, beans, seeds, nuts, flowers, leaves, cereal grains and tea [25,26,27]. Recent studies have reported that besides proanthocyanins there are other antioxidant compounds in red rice, such as γ-oryzanol, tocotrienols, tocopherols, carotenoids, flavones, flavonols and phenolic compounds, respectively [21,28]. Previously, proanthocyanidins extracted from persimmon peel and red wine have been shown to have cardioprotective effects, anti-oxidative and anti-diabetic [29,30]. Accumulating evidence in digestive diseases has suggested that proanthocyanidins-rich extract from natural products improves the gut barrier function, and attenuates the severity of inflammation conditions in experiments with IBD [31,32,33]. Sheng et al. [34] have previously demonstrated that the supplementation of grape seed proanthocyanidins extract (GSPE) reduces inflammatory cytokines and oxidative stress, maintains gut barrier integrity and improves intestinal microbiota compositions in DSS-induced colitis. Similarly, the administration of GSPE alleviates 2,4,6-trinitrobenzene sulfonic acid (TNBS)-induced colitis in rats by suppressing inflammatory responses. GSPE also inhibits the TNBS-induced inflammation in recurrent colitis by inactivating the nuclear factor-kappa B (NF-κB) pathway [35]. In the human study, dietary intake with proanthocyanidins increased mucus production, improved intestinal healing and reduced the frequency of bowel movements in UC patients [36].

Crude ethanolic extract from red rice has been shown to have an anti-invasion effect in MDA-MB-231 cancer cells [19]. Another study found that the polar fraction of red rice extract has a high content of proanthocyanidins, and exhibits anti-inflammatory effects by inhibiting inflammatory responses in lipopolysaccharide (LPS)-stimulated inflammation in RAW 264.7 cells [18]. In addition, PRRE not only inhibited cancer cell invasion, but also promoted tumor necrosis factor-alpha (TNF-α)-induced cell death [37]. However, the anti-colitis effect of PRRE has never been investigated and reported. In the present study, we hypothesize that PRRE alleviates the severity of clinical manifestations and suppresses the inflammatory responses of colitis induced by DSS, similar to the efficacy of the clinical drug sulfasalazine.

## 2. Materials and Methods

### 2.1. Preparation of Proanthocyanidins-Rich Red Rice Extract (PRRE)

Red jasmine rice (*Oryza sativa* L.) was obtained from the cultivation region in the Dok Khomtai District (Phayao, Thailand). Red jasmine rice was extracted following a previous study [19]. Approximately 1 kg of whole grains of red rice was mashed, and the powder was then soaked in ethanol (70%) with a shaker at room temperature for 12 h. After evaporation and lyophilization, the yield of crude extract was 1.053%. The ethanol extract was subsequently partitioned via liquid-liquid extraction with hexane, dichloromethane and ethyl acetate, with the aqueous residue phase yielding about 70% of the starting crude extract. Each fraction was obtained by lyophilization and stored at −20 °C until further study.

### 2.2. Measurement of Total Phenolic Content

Each fraction’s total phenolic content (TPC) was measured using a modified colorimetric Folin-Ciocalteu method [38]. Briefly, an extract sample of 20 μL was added with 100 µL of Folin-Ciocalteu’s reagent, and incubated in the dark at room temperature for 3 min. Then, 80 µL of 7.5% (*w*/*v*) sodium carbonate was added to the mixture and kept in the dark at room temperature for 30 min. The absorbance of all samples was determined at 765 nm. The TPC was calculated based on a standard curve of gallic acid, and expressed as milligrams of gallic acid equivalents per dry weight of the extract (mg GAE/g extract). The extract samples were assayed in triplicate.

### 2.3. Measurement of Total Proanthocyanidins Content

The total proanthocyanidin content (TPAC) in each fraction of red rice extract was determined using a vanillin assay [19]. In brief, each partitioned fraction was added with a sulfuric acid/methanol solution and 1% vanillin. The mixture was then incubated at 30 °C for 15 min and measured for absorbance at 490 nm by spectrophotometry, compared with a standard of catechin. TPAC was recorded as milligram catechin equivalents per gram of extract (mg CE/g extract).

### 2.4. High-Performance Liquid Chromatography (HPLC) Analysis

The constituents of proanthocyanidin monomers in PRRE were determined using the HPLC system. Briefly, 10 mg of PRRE was hydrolyzed with 1.0 M HCl and heated in boiling water for 30 min. The hydrolysate was then re-dissolved in MeOH. The hydrolysate was subsequently re-dissolved in a 1.0% (*v*/*v*) trifluoroacetic acid solution. The constituents of proanthocyanidin monomers in PRRE were determined using the HPLC system, according to a previous report [39], with slight modifications. The identification of flavan-3-ol monomers, including gallic acid, gallocatechin, epigallocatechin, catechin, caffeine, epicatechin, epigallocatechin gallate, gallocatechin gallate and epicatechin gallate was analyzed using the HP 1200 series liquid chromatography system (Agilent Technologies, Santa Clara, CA, USA). An aliquot of the hydrolysate sample was subjected to a Phenomenex C18 core-shell column. The isocratic elution of the mobile phase, which consisted of 0.1% acetic acid in acetonitrile and 0.1% acetic acid in the water, was set at 1.0 mL/min. The injection volume was 10 µL and the detection wavelength was 278 nm.

### 2.5. Animals and Experimental Design

C57BL/6 male mice (six-week-old, 23.33 ± 1.91 g) were obtained from the Nomura Company (Bangkok, Thailand). All mice were housed in a standard plastic case at room environmental control with a temperature of 25 ± 2 °C, humidity 50 ± 10% and a light-controlled facility (12 h light/dark cycle). The mice were fed with a standard laboratory pellet diet and given sterile water *ad libitum* for one week of adaptation. The experimental procedures were performed following protocols approved by the Institutional Animal Ethics Committee, University of Phayao, Thailand (Approval no. 5901040021).

**Figure 1:**
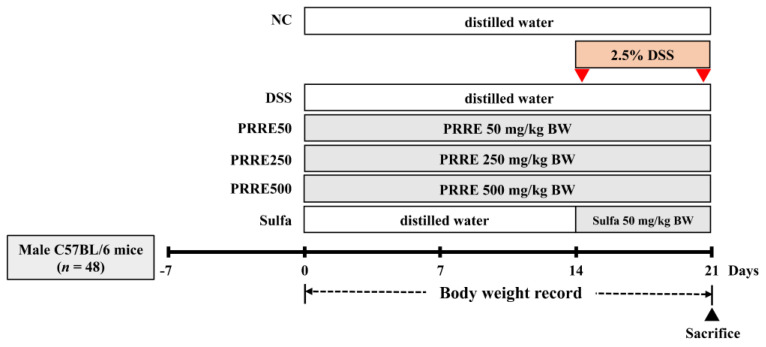
Overview of the experimental design for investigating the anti−colitis effect of PRRE against DSS−induced colitis in mice. DSS, dextran sulfate sodium; PRRE, proanthocyanidins rich red rice extract; sulfa, sulfasalazine.

After adaptation, mice were randomly allocated into six groups (*n* = 8/group). The experimental groups used in this study were as follows: the NC group was treated with a vehicle; the DSS group received DSS alone; the PRRE50 group received DSS and PRRE at 50 mg/kg/day; the PRRE250 group received DSS and PRRE at 250 mg/kg/day; the PRRE500 group received DSS and PRRE at 500 mg/kg/day; and the sulfa group received DSS and sulfasalazine at 50 mg/kg/day. The NC and the DSS groups were orally given distilled water as a vehicle throughout the experiment. The treatment groups were orally administered different doses of PRRE for 21 days. The doses of PRRE were selected as previously described [34,39]. Acute colitis of mice was subsequently induced by giving 2.5% DSS (*w*/*v*) (36–50 kDa, MP Biomedicals, Solon, OH, USA) in drinking water during the last seven days of the experimental period [40,41,42,43]. The reference group was treated with sulfasalazine (purity > 98%, Sigma Aldrich, St. Louis, MO, USA) at 50 mg/kg during the DSS induction [44]. The PRRE was prepared and mixed in distilled water, while sulfasalazine was prepared in 0.5% carboxymethyl cellulose. The body weight of mice was observed daily. At the end of the experiment, all mice were assessed for DAI, and were then euthanized by CO_2_ asphyxiation. Blood samples were immediately collected from the cardiac puncture into the heparin-contained syringe and kept on ice. Blood was then centrifuged at 3000 rpm for 30 min and plasma was stored at −80 °C until further analyzed. In addition, the colon was removed, opened longitudinally and rinsed with ice-cold phosphate-buffered saline. Each mouse’s colon length was measured and photographed. The small piece of the distal colon was subjected to histological evaluation. The experimental schedule is illustrated in Figure 1.

### 2.6. Assessment of Disease Activity Index

The disease activity index (DAI) scoring was determined based on clinical symptoms, including loss of body weight, stool consistency and blood in the stools [45]. The DAI was calculated from the average summation of scores of a percentage of weight loss, stool consistency and gross bleeding divided by 3 (Table 1). The occult blood was evaluated using the Hema-Screen^®^ Lab Pack for fecal occult blood (EKF Diagnostics, Boerne, TX, USA).

### 2.7. Histopathological Analysis of Colon Tissues

The distal colon sample was fixed overnight in a 10% buffered formalin solution and embedded in paraffin. The sections (5 μm) were stained with hematoxylin and eosin (H&E). Each section was observed under a light microscope (BX43, Olympus Corporation, Tokyo, Japan) and graded by a pathologist blind to the experimental protocol. The scoring of pathohistological change was graded in each colon specimen described previously [43,44], including the extent of erosion or ulceration (0–3), the area affected by inflammation (0–4), the extent of lymphoid follicle aggregate (0–3), edema (0–3), crypt loss (0–4) and degree of inflammatory cells infiltration (0–3).

### 2.8. Measurement of Pro-Inflammatory CytokinePproductions

Plasma levels of TNF-α, IL-1β and IL-6 were measured in duplicate using the enzyme-linked immunosorbent assay (ELISA) kit assay (Bio Legend, San Diego, CA, USA) according to the manufacturer’s protocol. Briefly, a 96-well plate added 100 μL of Capture Antibody solution in all wells, and the plate was sealed and incubated overnight at 4 °C. The plate was incubated for 30 min at room temperature, followed by washing four times with wash buffer. Then, 1× Assay Diluent was added to all wells and 50 μL of diluted standard or sample was added to each well and determined in duplicate. After incubation for 2 h at room temperature with shaking, the plate was washed, followed by adding 100 μL of Mouse IL-6 Detection Antibody and incubating at room temperature for 1 h with shaking. The plate was then washed, 100 μL of Avidin-HRP solution was added, and it was incubated at room temperature for 30 min with shaking. After that, the plate was washed, and tetramethylbenzidine substrate was added to each well and kept in the dark for 15 min. The color change was determined using a microplate reader (Metertech, Taipei, Taiwan) at 450 nm.

### 2.9. Periodic Acid—Schiff Taining

Neutral mucins containing goblet cells were analyzed using periodic acid–Schiff (PAS) staining, as described in the previous study [46]. In brief, the distal colon sections were deparaffinized and rehydrated, followed by receiving a periodic acid treatment for 10 min. Slide sections were then washed with distilled water and stained with the Schiff reagent for 15 min. Next, slides were rinsed with running water for 10 min, followed by a counterstain with hematoxylin. Images and observations were performed under a light microscope (BX43, Olympus Corporation, Tokyo, Japan). The PAS staining score was graded as excellent to poor (0–10), based on the number of mucin-secreting goblet cells in the intestinal epithelium.

### 2.10. Statistical Analysis

All data from the animal experiment are expressed as the mean ± standard error of the mean (SEM). Statistical analysis was performed using the one-way analysis of variance (ANOVA), followed by Tukey’s post-*hoc test* for comparative analysis. Differences were considered statistically significant at *p* value < 0.05.

## 3. Results

### 3.1. Total Phenolic Content and Total Proanthocyanidins Content of Fractionated Extracts FromRed Rice

Phenolics and proanthocyanidins have been reported as abundant compounds in red rice, with high anti-oxidative and anti-inflammatory actions [18,19]. Total phenolic content (TPC) and total proanthocyanidins content (TPAC) in each fraction of red rice extracts were determined. The results demonstrated that the aqueous fraction had the highest TPC and TPAC (Table 2). Therefore, the aqueous fraction was selected as PRRE for testing the anti-colitis effect on the DSS-induced colitis mouse model.

### 3.2. Identification of Proanthocyanidin Monomeric Units in PRRE

**Figure 2:**
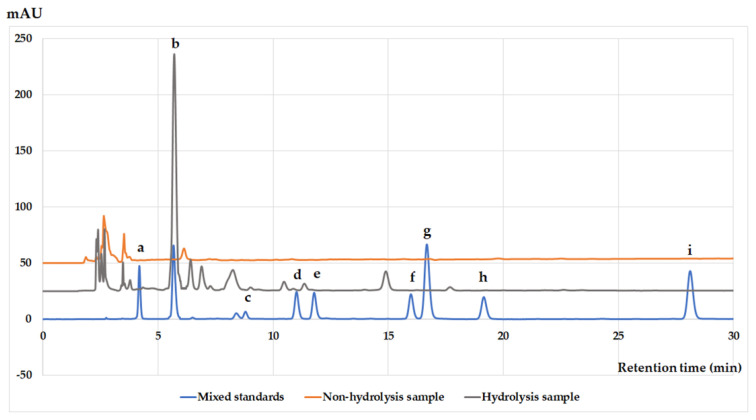
HPLC chromatographic separation (using UV detection at 278 nm) of seven catechins including gallic acid and caffeine in a standard mixture (blue line) compared to the non−acid hydrolyzed PRRE (orange line) and acid hydrolyzed PRRE (gray line). Note Gallic acid (a), Gallocatechin (b), Epigallocatechin (c), Catechin (d), Caffeine (e), Epicatechin (f), Epicallocatechin gallate (g), Gallocatechin gallate (h), Epicatechin gallate (i).

The extract was subjected to acid hydrolysis and analyzed by an HPLC system to identify the monomeric units of proanthocyanidins in PRRE (Figure 2). Commercial gallic acid (RT = 4.182), gallocatechin (RT = 5.569), epigallocatechin (RT = 8.794), catechin (RT = 11.012), caffeine (11.778), epicatechin (RT = 15.983), epigallocatechin gallate (RT = 16.675), gallocatechin gallate (RT = 19.150) and epicatechin gallate (RT = 28.118) were used as the standard (blue line). Before hydrolysis of PRRE, its HPLC chromatogram showed no monomeric units of proanthocyanidins (orange line), while the HPLC chromatogram of acid-hydrolyzed PRRE demonstrated the presence of mostly gallocatechin and slightly catechin (gray line). Thus, our result indicated that proanthocyanidins in PRRE is mainly composed of oligomeric gallocatechin.

### 3.3. PRRE Alleviates Clinical Manifestations in DSS-Induced Colitis

**Figure 3:**
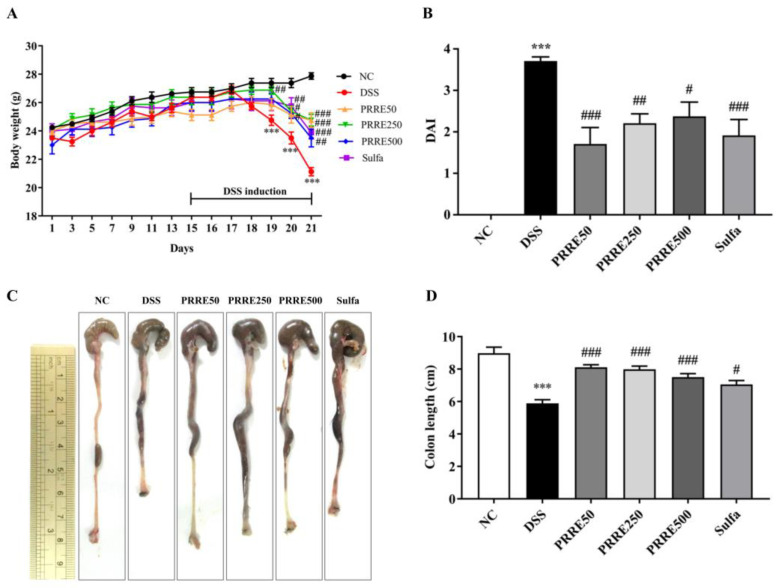
The effects of PRRE on clinical manifestations in DSS−induced colitis. The body weight of the animal (**A**), DAI scores (**B**), Gross appearances of the colon from each group (**C**) and Colon length (**D**). Data are expressed as mean ± SEM (*n* = 8). *** *p* < 0.001 vs. NC group, ^#^ *p* < 0.05, ^##^ *p* < 0.01 and ^###^ *p* < 0.001 vs. DSS group. DSS = dextran sulfate sodium, DAI = disease activity index, NC = normal control mice treated with the vehicle, DSS = mice treated with DSS alone, PRRE50 = DSS mice treated with PRRE at 50 mg/kg/day, PRRE250 = DSS mice treated with PRRE at 250 mg/kg/day, PRRE500 = DSS mice treated with PRRE at 500 mg/kg/day and sulfa = DSS mice treated with sulfasalazine at 50 mg/kg/day.

This study used the DSS-induced colitis model to investigate the anti-colitis effect of PRRE compared to the sulfasalazine in-*vivo* study. In the present study, oral administration of PRRE for 14 days before DSS administration did not show any sign of mortality and toxicity in mice. Mice were then given 2.5% DSS in drinking water for the last seven days of the experiment simultaneously with deionized water (DI), PRRE or sulfasalazine. The body weight significantly gradually decreased after DSS induction on days 5 to 7 in the DSS group, compared to the normal control (NC) group. In contrast, all doses of PRRE and sulfasalazine remarkedly ameliorated DSS-induced reduction of body weight (Figure 3A). Furthermore, DSS-treated mice exhibited severe clinical symptoms, including body weight loss (%), diarrhea and bloody stools, resulting in the highest score of the disease activity index (DAI) (Figure 3B). However, the DAI was significantly reduced in all doses with PRRE and sulfasalazine groups compared to DSS group (Figure 3B). In addition, the shortening of the colon is related to the severity of colitis. In this study, treatment with PRRE, particularly 50 mg/kg/day, was more effective than sulfasalazine in preventing colon shortening induced by DSS (Figure 3C,D). These findings suggest that all doses of PRRE and sulfasalazine alleviated the severity of colitis caused by DSS, and that 50 mg/kg of PRRE had the highest efficacy.

### 3.4. PRRE Prevents DSS-Induced Histopathological Damage

**Figure 4:**
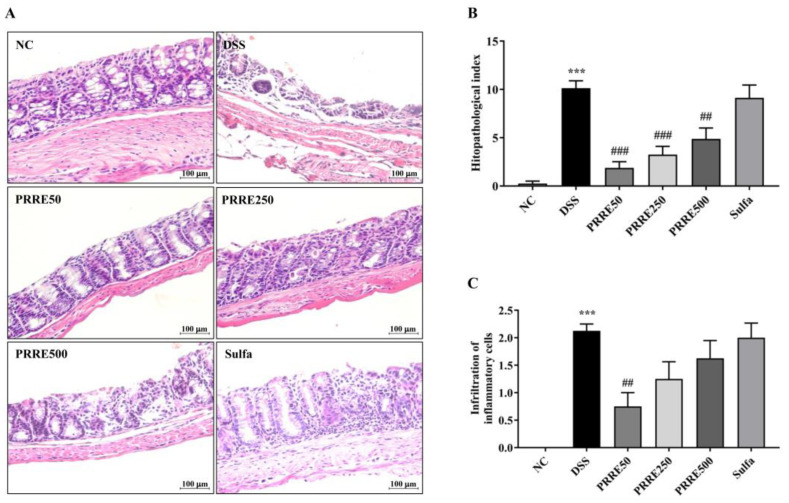
The effect of PRRE on histopathological damage in the colon induced by DSS. Histological images of distal colonic tissues stained with H&E after DSS induction for seven days (×200 magnification, scale bar = 100 μm) (**A**), Histopathological index (**B**) and Infiltration of inflammatory cells (**C**). Data are expressed as mean ± SEM (*n* = 8). *** *p* < 0.001 vs. NC group, ^##^ *p* < 0.01 and ^###^ *p* < 0.001 vs. DSS group. NC = normal control mice treated with the vehicle, DSS = mice treated with DSS alone, PRRE50 = DSS mice treated with PRRE at 50 mg/kg/day, PRRE250 = DSS mice treated with PRRE at 250 mg/kg/day, PRRE500 = DSS mice treated with PRRE at 500 mg/kg/day and sulfa = DSS mice treated with sulfasalazine at 50 mg/kg/day.

Histological examination of the colon tissue from normal control mice showed intact colonic architecture with no damage. The DSS-treated mice showed severe chronic active inflammation with ulceration. Disruption of colon crypts was identified among edematous stroma with marked inflammatory infiltration. However, these changes were improved by PRRE (Figure 4A). In addition, the DSS group had a significantly higher histopathological index and worse degree of inflammatory cell infiltration than the NC group (Figure 4B,C). On the other hand, all doses of PRRE treatment groups, but not in the sulfasalazine group, efficiently decreased the histopathological index and severity of overall inflammation compared with the DSS group. These results suggest that PRRE treatment, particularly a dose of 50 mg/kg/day, had the greatest effectiveness in attenuating colonic tissue damage induced by DSS.

### 3.5. PRRE Attenuates the Production of Proinflammatory Cytokines in DSS-Induced Colitis

**Figure 5:**
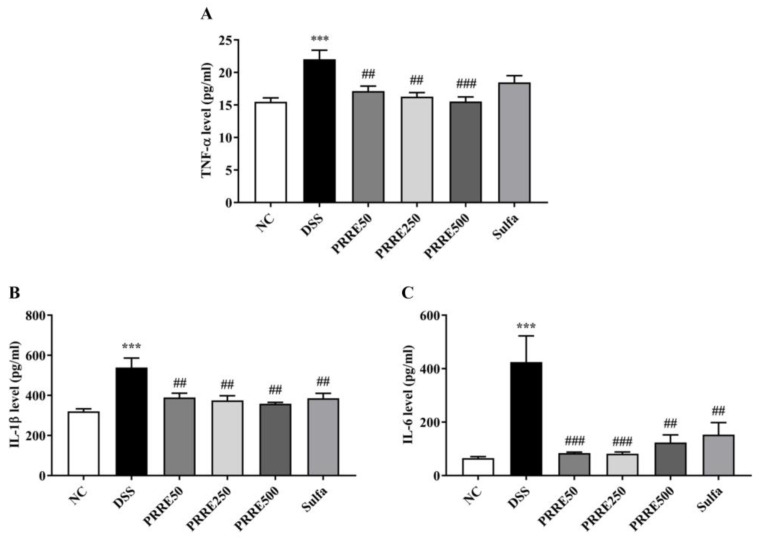
The effects of PRRE on productions of pro-inflammatory cytokine induced by DSS. The plasma levels of TNF-α (**A**), IL-1β (**B**) and IL-6 (**C**). *** *p* < 0.001 vs. NC group, ^##^ *p* < 0.01 and ^###^ *p* < 0.001 vs. DSS group. NC = normal control mice treated with the vehicle, DSS = mice treated with DSS alone, PRRE50 = DSS mice treated with PRRE at 50 mg/kg/day, PRRE250 = DSS mice treated with PRRE at 250 mg/kg/day, PRRE500 = DSS mice treated with PRRE at 500 mg/kg/day and sulfa = DSS mice treated with sulfasalazine at 50 mg/kg/day.

PRRE has recently been reported to suppress the inflammatory responses in LPS-stimulated inflammation in macrophage cells [18]. In this study, our results demonstrated that productions of TNF-α, IL-1β and IL-6 in DSS-treated mice were significantly higher than those in the normal mice. By contrast, all doses of PRRE treatment significantly diminished the production of TNF-α, IL-1β and IL-6 in plasma levels in an independent manner. In addition, sulfasalazine treatment reduced IL-1β and IL-6, but not TNF-α, as shown in Figure 5A–C. Thus, our results suggest that the efficacy of PRRE was remarkably higher than sulfasalazine in suppressing the production of pro-inflammatory cytokines induced by DSS.

### 3.6. PRRE Prevents Mucin-Secreting Goblet Cells in DSS-Induced Colitis

**Figure 6:**
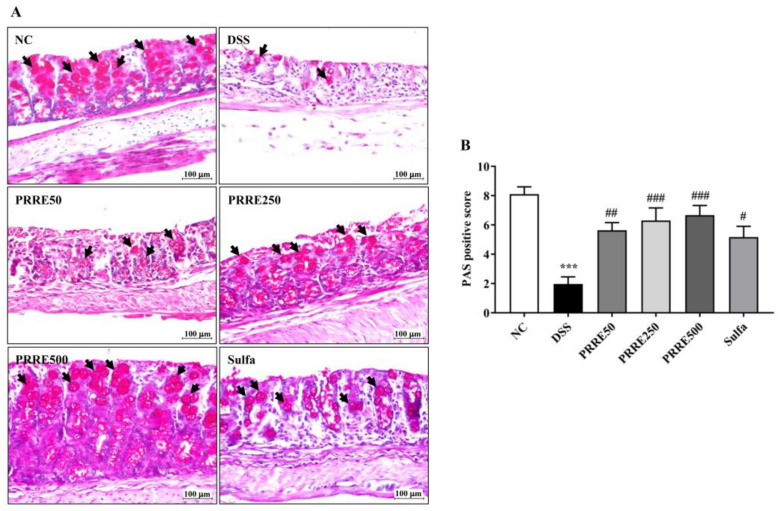
The effects of PRRE on mucin-secreting goblet cells in DSS-induced colitis. Representative picture of PAS staining in the colon tissue (×400 magnification, scale bar = 100 μm) (**A**). Black arrows indicate the mucin-secreting goblet cells and PAS staining scores (**B**). *** *p* < 0.001 vs. NC group, ^#^ *p* < 0.05, ^##^ *p* < 0.01 and ^###^ *p* < 0.001 vs. DSS group. PAS = Periodic acid–Schiff, NC = normal control mice treated with the vehicle, DSS = mice treated with DSS alone, PRRE50 = DSS mice treated with PRRE at 50 mg/kg/day, PRRE250 = DSS mice treated with PRRE at 250 mg/kg/day, PRRE500 = DSS mice treated with PRRE at 500 mg/kg/day and sulfa = DSS mice treated with sulfasalazine at 50 mg/kg/day.

We determined whether PRRE affects mucin-secreting goblet cells in DSS-induced colitis using periodic acid–schiff (PAS) staining. The NC group indicated a higher abundance of goblet cells (black arrows) in the colon. On the other hand, the mucus layer and goblet cells were significantly diminished in the DSS group compared to the NC group. Interestingly, the PRRE treatment remarkably improved mucus in colonic goblet cells in DSS-induced colitis in a dose-dependent manner, and similarly affected the sulfasalazine treatment (Figure 6A,B).

## 4. Discussion

Proanthocyanins derived from red rice extracts have been reported to cause potent anti-inflammatory activity in-*vitro* experiments [18]. This study examined the anti-colitis effect of PEER against the DSS-induced colitis mouse model, and compared it to sulfasalazine treatment. Our significant findings provide the first scientific evidence that PRRE treatment effectively alleviates the severity of clinical symptoms of colitis, reduces histopathological damage and infiltration of inflammatory cells in the colon, suppresses pro-inflammatory cytokine productions, and preserves mucin-secreting goblet cells in DSS-induced colonic inflammation in mice. It is interesting to note that 50 mg/kg/day of PRRE treatment had higher efficacy than the sulfasalazine treatment.

Proanthocyanidins are oligomeric or polymeric flavan-3-ols from flavonoid biosynthesis that consist mainly of catechin, epicatechin, gallocatechin and epigallocatechin units. Our results reveal that the main bioactive compound separated from red rice was identified as oligomeric gallocatechin. This finding was consistent with the previous study that flavan-3-ol monomers, including catechin, epicatechin, gallocatechin and epigallocatechin, were found in red rice proanthocyanidins-rich extracts [47]. The anti-colitis effect is strongly associated with antioxidant and anti-inflammatory activities of phenolics, flavonoids and particularly proanthocyanidins from grape seeds [13,33,34]. It was reported that proanthocyanidins are mainly found as bioactive phytochemicals in red and black rice, but not in white grains [48]. Whole grains of red rice exhibit high antioxidant capacity, due to the high content of phenolics and proanthocyanidins [48,49]. This finding is consistent with previous studies, indicating that red rice contains substantial amounts of phenolic and proanthocyanidins, which have a high potential for antioxidant and anti-inflammatory activities.

The chemical colitis model, DSS or TNBS, has been widely used to induce intestinal mucosal damage in in-*vivo* studies of acute and chronic colitis [13,50,51]. DSS induces the immunological and histopathological responses to resemble the pathogenesis of IBD in humans [50]. This study found that mice treated with DSS continuously for 7 days successfully induced acute colitis. It is notable that DSS-treated mice showed severe clinical symptoms of colitis, as reflected by loss of body weight after the 5th day of DSS induction, altered stool consistency and bloody diarrhea, leading to high DAI scores. Moreover, DSS directly damaged the colonic mucosa, causing colon shortening and histopathological changes, including mucosal erosion, crypt loss, submucosal edema and the infiltration of inflammation cells. This change suggests that activation of immune cells in the colon results in the imbalance of cytokines regulated, which are initiation diffuse superficial inflammatory lesions [52]. Our results interestingly showed that oral administration with all doses of PRRE for 21 days, followed by DSS induction for the last 7 days, effectively alleviated the severity of clinical symptoms, reduced body weight loss and the DAI score, and restored colon length shortening, histopathological damage and infiltration of inflammatory cells into the colonic mucosal of colitis induced by DSS. These results reflect the cytoprotective and anti-ulcer effects of PRRE, and these findings are consistent with the previous studies. Oral administration of proanthocyanidins from grape seed extract (200 mg/kg/day) for seven days has been shown to prevent recurrent colitis challenged with TNBS in rats, as indicated by reducing inflammatory cell infiltration and oxidant damage, regulating inflammatory response and enhancing tissue repair [53]. In addition, the intake of GSPE with a dietary dose of 75 mg/kg and a pharmacological dose of 375 mg/kg for 15 days has been shown to prevent intestinal inflammation, oxidative stress production and preserved gut permeability in LPS-induced intestinal dysfunction in rats [54].

The intestinal mucus layer plays a critical role in preserving the gut barrier to protect against pathogenic microbe invasion or chemicals getting into the intestinal epithelium. This mucus is composed of mucin that is secreted from goblet cells at the base of the intestinal crypt [55]. Accumulative data have demonstrated that the IBD patients and chemical-induced colitis model are related to impaired goblet cell function and dysregulated mucin synthesis. DSS-induced colitis has been reported to decrease mucus thickness and increase gut permeability in the colon, resulting in commensal microbes penetrating and reaching intestinal epithelial cells. As a result, the infiltration of immune cells was stimulated, and led to the development of colitis [56]. We found that the PAS-positive score in the colon of mice given DSS and PRRE/sulfasalazine was higher than that of those given DSS alone. Another study showed that cranberry proanthocyanidins improve the destruction of the gut barrier resulting from elemental enteral nutrition by enhancing the morphology and function of the mucus layer [57]. Thus, PRRE could prevent goblet cell loss and protect the disruption of the mucus layer from DSS-induced colonic inflammation.

Increasing evidence has demonstrated that productions of pro-inflammatory cytokines TNF-α, IL-1β and IL-6 are augmented in IBD patients and chemical-induced colitis animal models [5,58]. Augmentation of pro-inflammatory cytokine releases in colonic inflammation leads to mucosal immune activation in epithelial cells. This is a major event that initiates and activates intestinal inflammation. The blocking of pro-inflammatory cytokine release is accordingly one of the targets for IBD therapeutics [59]. We found that the plasma levels of pro-inflammatory cytokines TNF-α, IL-1β and IL-6 were enormously increased in the DSS-treated mice. In contrast, oral administration with all doses of PRRE remarkedly decreased these cytokine levels. Our finding suggests that PRRE, particularly at a low dose, effectively attenuates the severity of colitis by suppressing pro-inflammatory mediators in DSS-treated mice. This result was similar to previous in-*vitro* studies, showing that PRRE exhibits anti-inflammatory properties. It inhibits pro-inflammatory cytokine expressions by suppressing inflammatory pathways, including AP-1, NF-κB and MAPK pathways, in LPS-induced macrophage cells [18]. Other studies have demonstrated that proanthocyanidins obtained from blackberries inhibit the inflammatory mediator in LPS-induced Raw 264.7 cells. In addition, Sicilian pistachio nut-rich polymeric proanthocyanidins fraction suppress inflammatory response by inhibiting the IL-6 and IL-8 releases, prostaglandin E2 production and cyclooxygenase (COX)-2 protein expression. It also reduces paracellular permeability and decreases NF-κB activation in IL-1β-stimulated Caco-2 cells [60]. In animal studies, oral administration of GSPE has been shown to attenuate TNBS-induced colitis in rats by downregulating inflammatory mediators, inhibiting inflammatory cell infiltration and oxidation damage, promoting tissue repair, decreasing IL-1β concentration, and increasing the production of anti-inflammatory IL-2 and IL-4, antioxidant enzyme activities and levels of glutathione [13,61]. In addition, diets that include food rich in anthocyanin/proanthocyanidins, such as black rice and purple potatoes, have been shown to provide a protective effect against inflammation and increased gut permeability, as well as enhanced colon functions through the regulation of gut microbiota [62,63].

Recent studies have reported that large amounts of consumption flavan-3-ol and proanthocyanidins are not absorbed well in the gastric and small intestine. In these studies, when they reached the colon, they were metabolized into low-molecular-weight phenolic compounds by the colonic microbiota, and were also absorbed by colonocytes [64,65]. These results indicate that the positive effect of proanthocyanidins obtained from natural products is directly involved in improving colon functions. Several studies have shown the varieties of beneficial effects of the proanthocyanidins-rich extract on human health, including regulation of glucose and lipid metabolisms, improvement of insulin sensitivity and anti-inflammatory activity [27]. Recent studies using chemical-induced colitis in an animal model have shown that the dietary supplementation of proanthocyanidins-rich extracts leads to strong protection against gut barrier dysfunctions [34,54] through modulating gut microbiota [64], inhibiting inflammatory responses, inhibiting NF-κB activation [61], reducing myeloperoxidase activity and increasing antioxidant enzyme activity [35]. Beyond proanthocyanidins, primarily red rice extract also contains other active compounds, including γ-oryzanol, catechin and vanillic acid, which also exert anti-oxidative and anti-inflammatory activity [18,19]. These bioactive compounds may synergistically affect proanthocyanidins in anti-inflammatory and anti-colitis activities in DSS-induced colitis mice. Our findings suggest that the intake of a low dose of PRRE could exert beneficial effects on gastrointestinal-related inflammation diseases and cancers. Since different kinds of rice are a staple food consumed mainly by Asian countries, pigmented rice such as purple rice, black rice and red rice should be encouraged for their health benefits and social-economic advantages. In addition, Reagan-Shaw et al. [66] showed a method for translation of does from an animal to a human study. Therefore, the doses of PRRE (50–500 mg/kg/day) used in this animal study correspond to the dose of 4.054–40.54 mg/kg/day, and the PRRE doses in humans should be 243–2430 mg/human weight 60 kg. Therefore, further study from this research work is required to achieve beneficial effects in clinical trials.

In addition, these effects were comparable to those obtained with sulfasalazine, the standard drug used for IBD treatment. Our results demonstrated that oral administration of 50 mg/kg of sulfasalazine for seven days ameliorated the severity of clinical symptoms, and partially suppressed releases of inflammatory cytokine IL-1β and IL-6. However, sulfasalazine treatment insufficiently prevented pathohistological changes and the infiltration of inflammation cells in DSS-received mice. Another study showed that a sulfasalazine (30 mg/kg) combination therapy had higher efficacy in alleviating UC in animal models than treatment with sulfasalazine alone [52,67]. These results suggest that PRRE may be combined with sulfasalazine to increase therapeutic efficacy, and decrease the side effects of sulfasalazine in IBD patients.

## 5. Conclusions

Proanthocyanidins are oligomeric or polyhydroxyflavan-3-ol that consist mainly of catechin, epicatechin, gallocatechin and epigallocatechin units. We have shown that the main bioactive compound, proanthocyanins, separated from red rice was oligomeric gallocatechin. Our animal study is the first finding showing that PRRE has potential preventative benefits on colonic inflammation induced by DSS in mice. It is suggested that PRRE is mainly responsible for the prevention of intestinal inflammation. The PRRE significantly ameliorates clinical symptoms, and histopathological damage suppresses pro-inflammatory cytokines and improves mucin-secreting goblet cells in the colon. Interestingly, the natural PRRE had a stronger preventative efficacy than the sulfasalazine drug treatment. Therefore, PRRE may be further developed as a potential health-beneficial product as a food supplement or a promising alternative agent for IBD management, and clinical trials in humans need to be established in further investigations.

## Figures and Tables

**Table 1:** Assessment of disease activity index scoring.

Score	Weight Loss (%)	Stool Consistency	Occult Blood
0	None	Normal stool, well form pellets	No rectal bleeding
1	1.0–5.0	-	-
2	5.1–10.0	Loose stools, pasty stools that do not stick to the anus	Hemoccult positive
3	10.1–15.0	-	-
4	>15.0	Diarrhea, liquid stools that stick to the anus	Visible gross bleeding

**Table 2:** Total phenolic content and total proanthocyanidins content of fractionated extracts from red rice extract.

Fractionated Extracts	TPC	TPAC
Ethanol	56.44 ± 2.48	20.64 ± 2.39
Hexane	29.91 ± 1.08	0.30 ± 0.10
Dichloromethane	30.78 ± 1.91	0.34 ± 0.12
Ethyl acetate	100.75 ± 3.60	0.83 ± 0.15
Aqueous	136.69 ± 1.83	38.42 ± 1.89

Data expressed as mean ± SD (*n* = 3). TPC = total phenolic content (mg GAE/g extract), TPAC = total proanthocyanidins content (mg CE/g extract).

## Data Availability

The authors confirm that the data supporting the findings of this study are available within the article, and from the corresponding author upon request.
